# Fluid compartments influence elastography of the aging mouse brain

**DOI:** 10.1088/1361-6560/acc922

**Published:** 2023-04-17

**Authors:** Gary R Ge, Jannick P Rolland, Wei Song, Maiken Nedergaard, Kevin J Parker

**Affiliations:** 1 Institute of Optics, University of Rochester, 480 Intercampus Drive, Box 270186, Rochester, NY 14627, United States of America; 2 Center for Translational Neuromedicine, University of Rochester Medical Center, 601 Elmwood Avenue, Box 645, Rochester, NY 14642, United States of America; 3 Department of Electrical and Computer Engineering, University of Rochester, 724 Computer Studies Building, Box 270231, Rochester, NY 14627, United States of America

**Keywords:** brain, aging, fluid dynamics, optical elastography, biphasic model, rheology

## Abstract

*Objective*. Elastography of the brain has the potential to reveal subtle but clinically important changes in the structure and composition as a function of age, disease, and injury. *Approach*. In order to quantify the specific effects of aging on mouse brain elastography, and to determine the key factors influencing observed changes, we applied optical coherence tomography reverberant shear wave elastography at 2000 Hz to a group of wild-type healthy mice ranging from young to old age. *Main results*. We found a strong trend towards increasing stiffness with age, with an approximately 30% increase in shear wave speed from 2 months to 30 months within this sampled group. Furthermore, this appears to be strongly correlated with decreasing measures of whole brain fluid content, so older brains have less water and are stiffer. Rheological models are applied, and the strong effect is captured by specific assignment of changes to the glymphatic compartment of the brain fluid structures along with a correlated change in the parenchymal stiffness. *Significance*. Short-term and longer-term changes in elastography measures may provide a sensitive biomarker of progressive and fine-scale changes in the glymphatic fluid channels and parenchymal components of the brain.

## 1. Introduction

Elastography techniques are increasingly capable of producing quantitative images of the biomechanical properties of the human body. Elastography applied to the brain is a relatively recent and promising avenue of research using magnetic resonance imaging, ultrasound, and optical coherence tomography (OCT) imaging systems in clinical and pre-clinical settings. An overarching goal is to discover sensitive biomarkers related to the viscoelastic properties of the brain (Hiscox *et al* 2016, 2018, 2021, Bigot *et al* 2018, Gerischer *et al* 2018, Munder *et al* 2018, Guo *et al* 2019, Murphy *et al* 2019, Arani *et al* 2021) that can be targeted for diagnosis. These previous studies demonstrate that there are changes in brain viscoelastic properties with age, injury, and disease. However, the fundamental mechanisms underlying these changes and their links to the tightly regulated brain vascular, perivascular, and glymphatic fluid systems, remain as questions to be systematically studied. Thus, the need for a detailed examination of key factors and mechanisms that influence brain stiffness in normal aging is still a major topic for research. This paper utilizes an advanced elastography technique and rheological model to measure brain stiffness versus age and fit the data to plausible changes in governing parameters. The key role of fluid channels, especially within the brain’s glymphatic system, is closely examined. This paper is organized to review the necessary equations for the biphasic rheological model, then describe the elastography measurements and present the results. The role of fluid channels and the global water content of the brain are of particular interest as to how they contribute to the strong stiffening of the mouse brain with advanced age.

## 2. Theory

In this section we summarize the main results from recent work on the biphasic (fluid/solid) microchannel flow model applied to the brain (Parker 2017, Ge *et al* 2022a). We derived the stress-strain behavior of the brain as a two-compartment model with a larger scale network representing the vascular and perivascular branching structures plus a smaller scale version representing the interstitial spaces, in particular the glymphatic system. The stress relaxation response of a macroscopic block of tissue is given by

\begin{eqnarray*}\begin{array}{l}{\sigma }_{SR}\left(t\right)={A}_{1}\left(\displaystyle \frac{{\mathrm{\Gamma }}\left[{a}_{1},\tfrac{t}{{\tau }_{{1}_{\max }}}\right]-{\mathrm{\Gamma }}\left[{a}_{1},\tfrac{t}{{\tau }_{{1}_{\min }}}\right]}{{t}^{{a}_{1}}}\right)\\ \,+\,{A}_{2}\left(\displaystyle \frac{{\mathrm{\Gamma }}\left[{a}_{2},\tfrac{t}{{\tau }_{{2}_{\max }}}\right]-{\mathrm{\Gamma }}\left[{a}_{2},\tfrac{t}{{\tau }_{{2}_{\min }}}\right]}{{t}^{{a}_{2}}}\right),\end{array}\end{eqnarray*}

where *A*_1_ represents the stiffness of the vascular and perivascular fractal branching structures and *A*_2_ the smaller scale interstitial/glymphatic structures, *a* is the power law exponent for each compartment, *τ*_max_ and *τ*_min_ are the largest and smallest time constants, respectively, associated with the network of fluid channels permeating the tissue, and Γ represents the upper-tailed Gamma function (Abramowitz and Stegun 1964). These parameters are conditioned by anatomical measures and are discussed in more detail in Ge *et al* (2022a). The complex Young’s modulus as a function of frequency is then derived by Laplace transform theory as the sum of two groups corresponding to the *A*_1_ and *A*_2_ terms above. However, a simplified form for the *A*_2_ function is possible and so our working approximation for the complex Young’s modulus is:

\begin{eqnarray*}\begin{array}{l}E\left(\omega \right)={A}_{1}{\left(-I\omega \right)}^{a}\left(\beta \left[\left(-I\omega {\tau }_{\min }\right),\left(1-a\right),0\right]\right.\\ \,\left.-\beta \left[\left(-I\omega {\tau }_{\max }\right),\left(1-a\right),0\right]\right)+\left({A}_{2}/{\tau }_{\min }^{{a}_{2}}\right)\left(1/a\right),\end{array}\end{eqnarray*}

where $\omega $ is the radial frequency of the shear waves employed in elastography, *I* is the imaginary unit, and *a* > 0 is the power law exponent linked to the distribution of fluid channel sizes. This approximation is valid for typical frequencies where $\omega \gg 1/{\tau }_{{\mathrm{\min }}},$ and the shear wave speed (SWS) as a function of frequency (the phase velocity and dispersion) can also be calculated from this quantity. In this equation, the *A*_2_ term is an asymptotic approximation to the beta function terms with very long *τ* time constants valid over the typical range of frequencies used in elastography. This is a result of the time constants within the glymphatic system being so long as to result in a nearly constant term over the typical range of brain elastography experiments above 10 Hz.

Next, we consider dilation or constriction of the fluid channels within either of the two compartments. It can be shown that this change can be treated simply with scale factors shifting both the magnitude and the time constants associated with the network of fluid channels.

If all the vessel radii are increased or decreased by a factor of ${r}_{2}=\chi r$ where $\chi > 1$ represents dilation and $\chi < 1$ represents constriction, then we find

\begin{eqnarray*}\begin{array}{l}{\tau }_{{2}_{\max }}=C/{\left(\chi {r}_{\min }\right)}^{1.5}={\left(1/\chi \right)}^{1.5}{\tau }_{\max }\,{\mathrm{and}}\\ {\tau }_{{2}_{\min }}=C/{\left(\chi {r}_{\max }\right)}^{1.5}={\left(1/\chi \right)}^{1.5}{\tau }_{\min },\end{array}\end{eqnarray*}

where *C* is a constant based on material properties and the exponent of 1.5 is a plausible value derived from anatomical measures of the brain as explained in Ge *et al* (2022a).

We find that a general rule for the change in stiffness and particularly stress relaxation as a function of overall change in fluid vessels within a biphasic tissue is given by

\begin{eqnarray*}{\sigma }_{SR2}\left(t\right)=\left(\displaystyle \frac{{A}_{0}}{{\chi }^{1.5a}}\right)\displaystyle \frac{\left({\mathrm{\Gamma }}\left[a,t/{\tau }_{{2}_{\max }}\right]-{\mathrm{\Gamma }}\left[a,t/{\tau }_{{2}_{\min }}\right]\right)}{{t}^{a}},\end{eqnarray*}

where ${\tau }_{{2}_{{\mathrm{\max }}}}$ and ${\tau }_{{2}_{{\mathrm{\min }}}}$ are given by equation (3). Thus, equations (3) and (4) describe the change in rheology as a function of fluid channel changes proportional to $\chi ,$ in an unconfined space. As a simplification, the leading term of $1/{\chi }^{1.5a}$ can be considered the dominant factor, showing a direct effect where small amounts of dilation create a softening of the tissue. This term directly affects the complex modulus, introducing the *χ* parameter into equation (2), and where the $\tau $ time constants have been modified by equation (3).

Now consider the case where the elastic properties of the cellular structures change, without any alteration of vessel diameters. Electro-chemical effects in different cells, axons, dendritic spines, cell membranes, and actin filaments have been reviewed by Tyler (2012) and Barnes *et al* (2017). Functional stimuli may incite regional electro-chemical changes (Patz *et al* 2016). Furthermore, the intracellular water content, bound water, and components including myelin and proteoglycans may not be a constant across the different ages, so the model’s baseline parenchymal stiffness may not be treated as a constant.

Again, assuming a baseline case of ${\sigma }_{S{R}_{b}}\left(t\right),$ then if ${E}_{2}={\chi }_{E}E$ as a change in the elastic matrix by factor of ${\chi }_{E},$ we can map the resulting changes through the transformation rules (Parker 2017) and we find that:

\begin{eqnarray*}{\sigma }_{SR2}\left(t\right)=\displaystyle \frac{{\chi }_{E}^{\left(1-a\right)}{A}_{0}\left({\mathrm{\Gamma }}\left[a,t/{\tau }_{{2}_{\max }}\right]-{\mathrm{\Gamma }}\left[a,t/{\tau }_{{2}_{\min }}\right]\right)}{{t}^{a}}.\end{eqnarray*}

where ${\tau }_{{2}_{{\mathrm{\max }}}}={\tau }_{{\mathrm{\max }}}/{\chi }_{E}$ and ${\tau }_{{2}_{{\mathrm{\min }}}}={\tau }_{{\mathrm{\min }}}/{\chi }_{E}.$

Thus, an increase in $E$ (${\chi }_{E}> 1$) translates into two effects: an increase in the overall stress relaxation force by a factor of ${\chi }_{E}^{\left(1-a\right)},$ and a down-shifting of time constants by a factor of ${1/\chi }_{E},$ which in some cases produce a slight ‘softening’ effect.

Now summarizing and combining these trends, the leading terms in the equations for the complex modulus can be written as

\begin{eqnarray*}{E}_{2}=\displaystyle \frac{{E}_{0}{\chi }_{E}^{\left(1-a\right)}}{{\chi }^{\left(1.5a\right)}},\end{eqnarray*}

where *E*_2_ is the altered modulus as $\chi $ and ${\chi }_{E}$ are varied around a reference point of 1 corresponding to a reference modulus of *E*_0_. This simplified equation assumes the effect on changing the *τ*_max_ and *τ*_min_ is less significant but serves as a straightforward approximation. Another hypothesis to be re-examined later is that ${\chi }_{E}$ can be approximated as inversely proportional to ${\left({\chi }^{3}\right)}^{2}.$ The ${\chi }^{3}$ term reflects a strong correlation with increasing or decreasing volume of water in the fluid spaces of the brain. The square term captures the measured dependence of biomaterial (elastic phantom) moduli as a function of water/solid content, although in the case of gelatin phantoms there is some disagreement as to the dependence being linear or square (Hall *et al* 1997, Zhang *et al* 2011, Nguyen *et al* 2014). Assuming the square dependence and combining the terms yields an approximate expression for long-term changes of the complex modulus *E*_2_ in the brain from some nominal value ${E}_{0},\,$where *E*_2_ is given as:

\begin{eqnarray*}{E}_{2}=\displaystyle \frac{{E}_{0}}{{\chi }^{\left(1.5a+\left(1-a\right)6\right)}}=\displaystyle \frac{{E}_{0}}{{\chi }^{\left(6-4.5a\right)}},\end{eqnarray*}

and, for example, if *a* = 0.05, then the denominator term is ${\chi }^{5.75},$ a very strong dependence of the modulus with variations in fluid content.

## 3. Methods

### 3.1. Animal preparation

Thirty-five wild-type mice (C57BL/6, ranging from 2.5 to 30.6 months of age, 22 male/13 female, Charles River Laboratories, Wilmington, MA, USA) were scanned with 3D reverberant shear wave field optical coherence elastography (Rev3D-OCE) during anesthetized (mimicking sleep) states (Ge *et al* 2022a). Cranial window surgeries were performed where 5 mm diameter glass windows replaced a portion of the skull near the center but leaning towards the right hemisphere (Mestre *et al* 2020). An agarose gel solution (1.1%) was used to adhere the glass window to the mouse skull/brain interface, and the *dura mater* was left intact. Rev3D-OCE scans were performed 15 min post-anesthesia (ketamine-xylazine) for the sleep state. Upon scan completion, the mice were euthanized, and the brains were extracted to measure brain water content. The wet and dry weights in grams (${w}_{{\mathrm{wet}}}$ and ${w}_{{\mathrm{dry}}},$ respectively) were measured at time of euthanasia and after 72 h incubation at 65 °C, respectively. Brain water content was computed as $\left[\left({w}_{{\mathrm{wet}}}-{w}_{{\mathrm{dry}}}\right)/{w}_{{\mathrm{wet}}}\right]\times 100 \% .$ Mice experiments were performed under protocols approved by the University of Rochester Committee on Animal Resources. Further details regarding related animal preparation are described by Ge *et al* (2022a).

### 3.2. Optical coherence elastography

**Figure 1.**
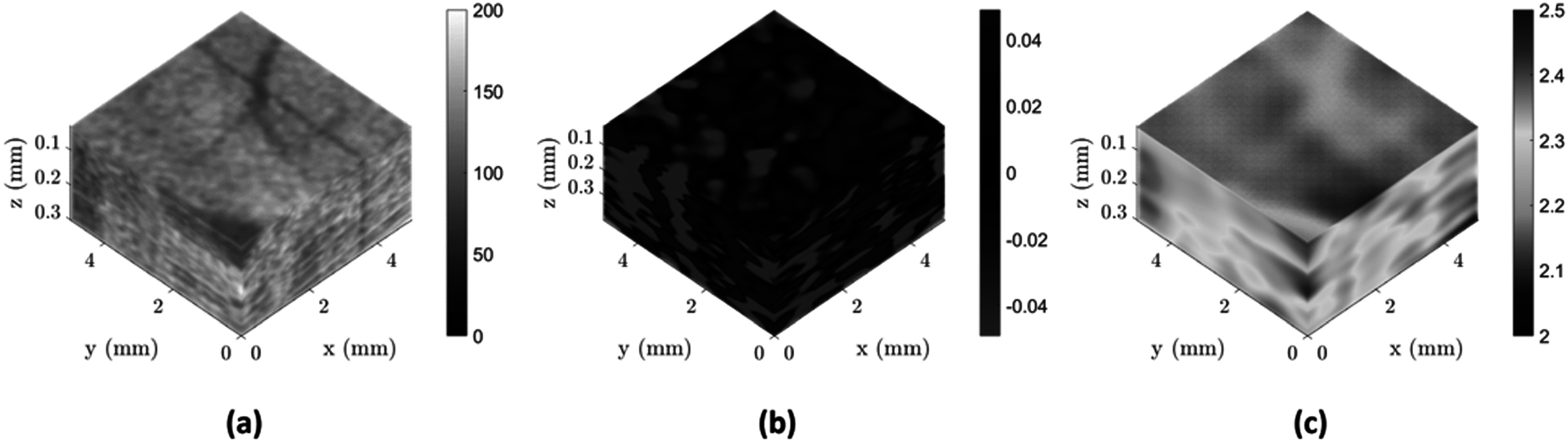
An example of elastography in the mouse brain using 2000 Hz reverberant shear waves. (a) 3D data set from the OCT scan of the anterior cortical brain (top) with a resolved blood vessel under the intact *dura mater*. (b) Instantaneous displacement patterns within the reverberant shear wave field, with red and blue colors indicating the direction positive or negative of the displacements. (c) Estimated SWSs within the 3D volume with some spatial variation corresponding to proximity to the blood vessel. Colorbar units are arbitrary grayscale in (a), arbitrary phase displacement in (b), and in m s^−1^ in (c).

As referenced above, Rev3D-OCE is implemented with a custom-built OCT and mechanical piezo-electric system, operated by LabVIEW (version 14, National Instruments, Austin, TX, USA). To summarize, the laser source is a swept-source laser (HSL-2100-HW, Santec, Aichi, Japan) with a center wavelength of 1310 nm and bandwidth of approximately 140 nm. The lateral resolution is estimated to be 20 *μ*m and the axial resolution to be 6 *μ*m in air. The field of view was adjusted to be approximately 5 × 5 mm. The mechanical piezo-electric system utilizes a custom 3D-printed ring that is 10 mm in diameter (8 mm aperture) and has eight points of contact to generate reverberant shear wave fields. The frequency of the piezo-electric device it set to be 2000 Hz. Synchronized M- and B-mode acquisition is used to acquire 4D data (3D space and time). In the scanned volumes of interest, local autocorrelations are performed with varying window sizes, which are proportional to the contours of B-mode intensities. Select frames in time are averaged to obtain the final 3D elastogram. Details on this methodology are adapted from previous studies (Zvietcovich *et al* 2019, Ge *et al* 2022b). All elastogram estimations and data processing are completed in MATLAB 2022b (Mathworks, Natick, MA, USA). One example brain study is shown in figure 1, covering from left to right the 3D scanned volume, the detected shear waves produced within the brain at 2000 Hz, and the estimated shear wave speed within the volume.

### 3.3. Data analysis

For each 3D elastogram, a mean SWS, ${c}_{s},$ in m s^−1^ is reported as the average within rectangular volumes that appear to be mostly brain parenchyma. The Young’s modulus can be computed via the formula

\begin{eqnarray*}E=3\rho {c}_{s}^{2},\end{eqnarray*}

where we assume the density $\rho $ of the medium to be 1000 kg m^−3^ or 1 g cm^−3^. The entire cohort of data points is summarized by plotting relevant measurements (age, measured SWS or Young’s modulus, and brain water content). Polynomial fitting along with coefficient of determination *R* ^2^ scores are reported for correlating trends. All data analysis is done using Python 3.11.1 (Python Software Foundation, Wilmington, DE, USA).

## 4. Results

**Figure 2.**
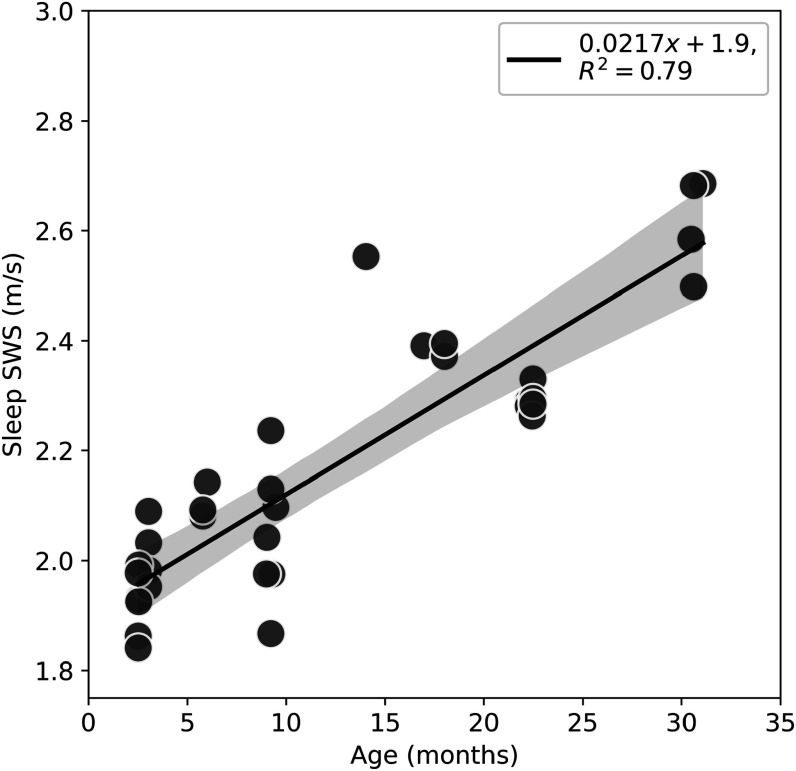
Shear wave speed (m s^−1^) measured in anesthetized wild-type mice cortical gray matter, *in vivo*, as a function of age in months. A linear regression line is included with the shaded area indicating a 99% confidence interval. The regression line has an *R* ^2^ of 0.79.

The shear wave speed (SWS) was found to be a strong function of age, increasing by a factor of nearly 30% over the measured span from youth to senescence (2.5 to 30.6 months). These results indicate that this strain of mice exhibits a pronounced trend whereby the brain stiffens with age (figure 2).

**Figure 3.**
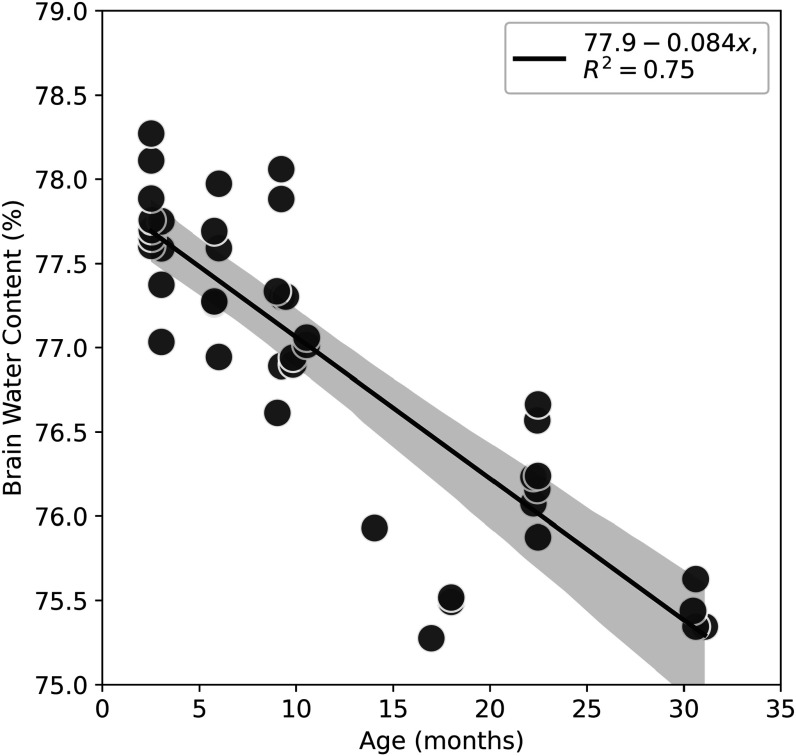
Percent water content measured as a function of age from hemispheres. Globally, the water content decreases with age, the linear regression is *R* ^2^ = 0.75.

Next, we examine the global fluid content of the brains as a function of age, based on conventional wet/dry weights of hemispheres. This is a particular interest given the biphasic nature of our rheological model. These results are shown in figure 3 and demonstrate a decreasing trend with age, showing higher water content in younger brains.

**Figure 4.**
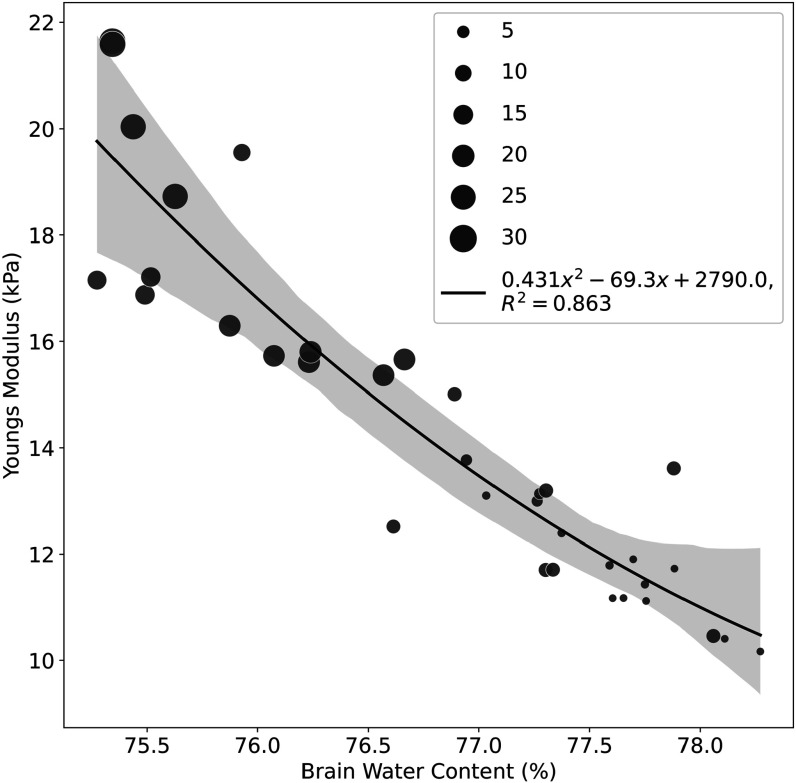
Measured stiffness (Young’s modulus in kPa) versus brain water content (% wet/dry weight) in wild type mice of different ages. Data point size is proportional to age in months. The younger mice (smaller circles) trend towards a higher fluid content and softer brains. The older mice (larger circles) trend towards drier and stiffer properties. The coefficient of determination *R* ^2^ is 0.86. The solid line is the second order polynomial fit with 99% confidence interval indicated as the shaded region.

Next, treating age as a parametric variable, we plot the stiffness as a function of water content in figure 4. In this graph, age is indicated by the size of the circles used, and a second order fit to the data is applied, with an overall *R* ^2^ of 0.86. This link is examined quantitatively in the model to determine plausible mechanisms underlying this trend.

Our model for the results above utilizes the simplified equations (6) and (7) with the following parameters: the reference point is taken arbitrarily as 77% water, corresponding to young adult mice in our samples, figures 3 and 4, with a modulus of 13.3 kPa. The percent of water is increased or decreased about this point by assuming all the increase or decrease is attributed to the glymphatic extracellular fluid compartment at roughly 10% of the overall fluid content:

\begin{eqnarray*} \% \,{\mathrm{water}}=67+10{\chi }^{3}.\end{eqnarray*}

**Figure 5.**
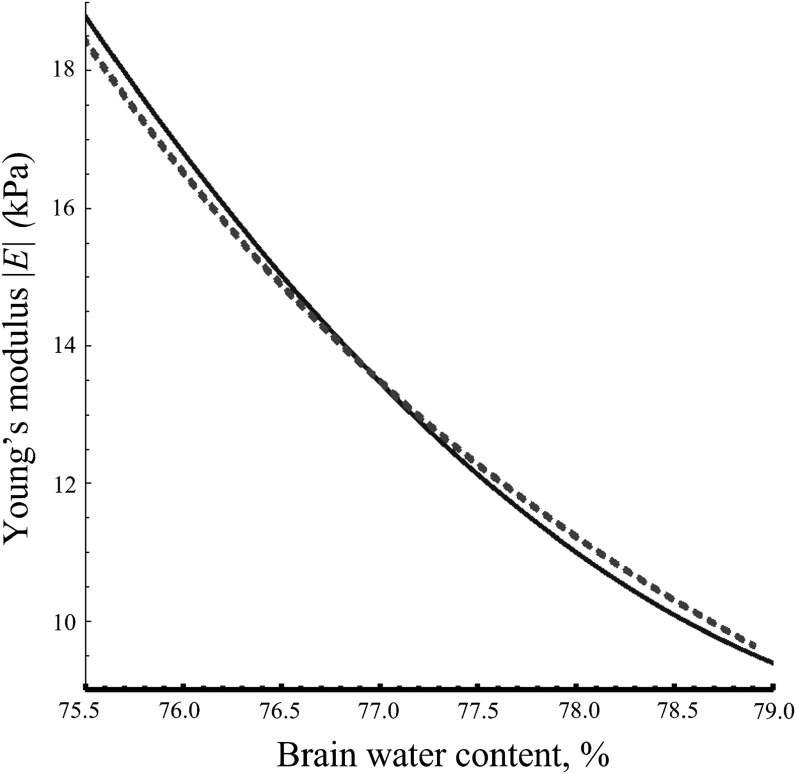
Vertical axis: magnitude of *E* at 2000 Hz in the brain using the microchannel flow model as described in the Theory section. Horizontal axis: brain percent water. Blue dashed line: model where the dilation parameter *χ* varies as the parametric variable between 0.94 < *χ* < 1.06 using equations (7) and (9). Red line: curve-fit of mouse brain data from figure 4. These demonstrate that fluid changes attributed to the glymphatic compartment and long-term changes in the elastic properties of the parenchymal matrix can combine to create pronounced changes in the stiffness of the brain.

This relation implies that the larger volumes of fluid in the intracellular spaces and the major blood vessels and cerebral spinal fluid (CSF) spaces are relatively constant across our sample space (O’Brien and Sampson 1965, Siegel *et al* 1999, Elkin *et al* 2010, Keep *et al* 2012, Jessen *et al* 2015, Reichel 2015, Gottschalk *et al* 2021). Then, for the modulus of *E* given by equation (7), assuming *a* = 0.05 as a benchmark, and *E*_0_ = 13.3 kPa, we can plot the modulus as a function of water content, with *χ* varying as the parametric parameter between 0.94 < *χ* < 1.06. This plot is shown in figure 5, along with a second order fit (red) representing the minimum mean squared error trend of all the data in figure 4.

## 5. Discussion

The overall set of measurements show clear trends with respect to the overall fluid content of the brains. As the water percent increases, the SWS and magnitude of the complex modulus (or ‘stiffness’) of the brain decrease. The microchannel flow model is capable of capturing these trends with relatively simple adjustment in parameters to account for dilation or constriction of the fluid channels. It was necessary for us to assume, for the conditions studied, that across the population the parenchymal elasticity was also dependent on the overall fluid content. In other words, this is not a simple vasoconstriction/vasodilation model but also includes a term reflecting a change in the underlying elastic modulus of the brain parenchyma correlated with age and overall percent of fluids within the brain, especially within the microchannels comprising the glymphatic drainage system. These assumptions are consistent with some earlier studies of aging brains (O’Brien and Sampson 1965, Keep *et al* 2012, Gottschalk *et al* 2021). The overall concept is illustrated in table 1. We note also that we tried several alternative models that failed to capture the significant trend in the elastography data with global brain water content. These are described in more detail in the appendix.

**Table 1.** Overview of model emphasizing key compartments. The key parameter $\chi $ represents a small change in radius of fluid channels from some reference value. Then ${\chi }^{3}$ is proportional to volume change in overall fluid within the fluid channels. Changes with aging can be modelled with principal dependence or correlation with changes in the fluid volume associated with the glymphatic system and with the elastic properties of the parenchymal matrix of the brain.

Microchannel flow model brain compartments	Change with age	Result
Parenchymal matrix	Water content decreases and stiffness increases ∼ (1/*χ* ^3^)^2^	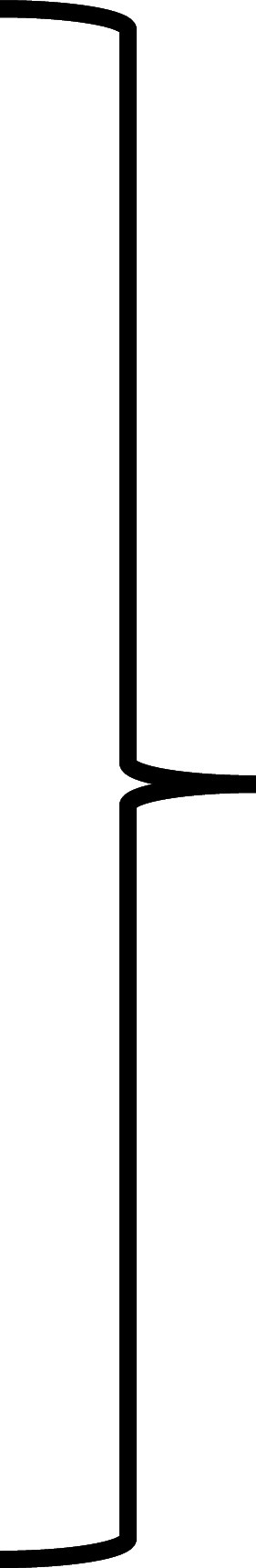 strong stiffening with drier,
Vascular/perivascular	Elasticity may increase, not included in model	older brains
Cerebral spinal fluid	No change in model	∼(1/*χ* ^6^)
Extracellular/glymphatic	Fluid volume decrease, causing elasticity increase ∼(1/*χ* ^1.5*a*^)	where *χ* < 1 in the aging brain

Since there is a wide spectrum of changes in the brain as a function of age, sorting through the dominant factors affecting stiffness is challenging and remains open for investigation. Just one factor, for example, that could play a role is the pronounced thickening of the basement membranes of the brain with age. The basement membranes are a thin but widely distributed layer within the blood-brain barrier and have been found to double in thickness with age in rodent brains. They thicken in response to mechanical stresses and, with age, may develop altered lipid, laminin, fibronectin, and other proteoglycan components (Ceafalan *et al* 2019, Reed *et al* 2019). This is one example of a morphological change accompanied by compositional changes that could create whole brain shifts in the baseline viscoelastic properties, since the thickness increases may affect the fluid channel dimensions ($\chi $), and the compositional changes may influence the composite stiffness of the elastic brain (${\chi }_{E}$), as supported by our model. Isolating these effects will require careful targeting and focus.

The comparison of our results with human brain magnetic resonance elastography (MRE) aging studies is complicated. There are a number of questions from MRE of the human brain that require clarification. In a recent review article (Arani *et al* 2021), the authors point out that a general consensus regarding the effects of aging on the brain of adult humans is that the brain will soften over time, however not all studies agree on the magnitude of this effect (Hiscox *et al* 2021, Coelho and Sousa 2022). Furthermore, some significant baseline parameters such as the relative stiffness of cortical grey matter versus cerebral white matter do not have a strong consensus across reports. We hypothesize that there are several factors in nominally healthy humans at age 80 that are different from the mouse brain at age 30 months, for example inflammatory or early-stage degenerative conditions that may be subclinical. Furthermore, the shear wave frequency difference in our mouse studies (2000 Hz) versus human studies (approximately 50 Hz is commonly used) can create complicated differences linked to viscoelastic dispersion effects that may not yet be well understood. Identifying key differences will require further comparisons.

Other MRI studies have linked diffusion estimates to microstructural alterations in the aging human brain including myelin and water (Billiet *et al* 2015, Beck *et al* 2021). It is possible that a multiparametric combination of these measures with elastography will help to improve the specificity of changes with respect to microstructural components.

As an aside for the rheology experts, it is important to note that our key model of complex stiffness, equation (2), is closely related to the well-known Kelvin–Voigt fractional derivative (KVFD) model. The leading term of ${\left(I\omega \right)}^{a}$ resembles the KVFD spring-pot (the fractional derivative damper term) and the constant asymptotic term for the glymphatic system resembles the parallel elastic spring in the KVFD model, which has found numerous uses in biomechanics (Parker *et al* 2019). The spring-pot (power law) behavior is recognizable when data are plotted on log-log graphs of stiffness versus frequency, the power law is observed as a straight-line dispersion curve over the frequencies where the ${\left(I\omega \right)}^{a}$ term dominates. This behavior is demonstrated clearly in the preponderance of brain measurements graphed by Forte *et al* (2017). Also, power law dispersion was noted in earlier mouse brain MRE studies by Clayton *et al* (2011).

The major improvement here is that our model explicitly links the parameters to anatomical measures of the vascular system and the glymphatic system, and then can account for rapid or long-term changes in these. This link to mechanisms within the brain helps to clarify the major factors that are captured in elastography of the brain and their diagnostic value.

Limitations of this work include the localized nature of the OCT elastography, within the cortical grey matter, and the single frequency of shear wave measurements, 2000 Hz. These should be expanded in future studies to obtain a fuller assessment of the regional dispersion properties as a function of age throughout the structures of the brain. Also of high interest for future work is a multifaceted study that would include local measures of the related changes in the aging mouse brain from a molecular to structural level of organization. These are known to include dysregulation of calcium homeostasis, loss of myelin (Schregel *et al* 2012, Weickenmeier *et al* 2016), and inflammatory responses (Radulescu *et al* 2021) as well as a variety of morphological changes including the doubling in thickness of the basement membranes. The influence of changes in cerebral blood volume with aging (Leenders *et al* 1990) on elastography also requires a careful assessment. The quantification of these along with fluid channel measures would lead to a more complete determination of the dominant factors setting the elastic properties of the aging brain. Finally, the distribution of age groups in our population was weighted toward younger mice due to common practices in colony management and commercial availability, and an equal distribution across ages was not feasible given the limited timeframe of the study along with the natural lifespan of this species. Larger samples of the group over 25 months would be helpful in confirming the trend line of elastography versus age, and these additions plus comparisons against other genotypes of mice models of brain disease are planned for future studies.

## 6. Conclusion

We have found a strong trend versus age in wild type mice where the cortical grey matter stiffness, as measured by shear waves at 2000 Hz, increases over time. The age effect produces an approximately 30% increase in shear wave speed (corresponding to a 70% increase in shear modulus) between the young 2.5 month old mice and older 30.6 month mice. Older brains are stiffer, and this is strongly correlated with the decreasing global measure of water content of the older brains. Our rheological model of the brain is based on the multiscale distribution of fluid channels throughout the brain. We find that the closest model matching the overall experimental trend allocates small changes in water content within the glymphatic system (smaller microchannels increase fluid resistance and stiffness) plus a correlated change to the parenchymal matrix (the drier and older brains are stiffer). With these combined effects, small shifts in the water content of compartments can replicate the overall trend of increasing stiffness with age and can be tracked by a plausible rheological model.

## Figures and Tables

**Figure A1.**
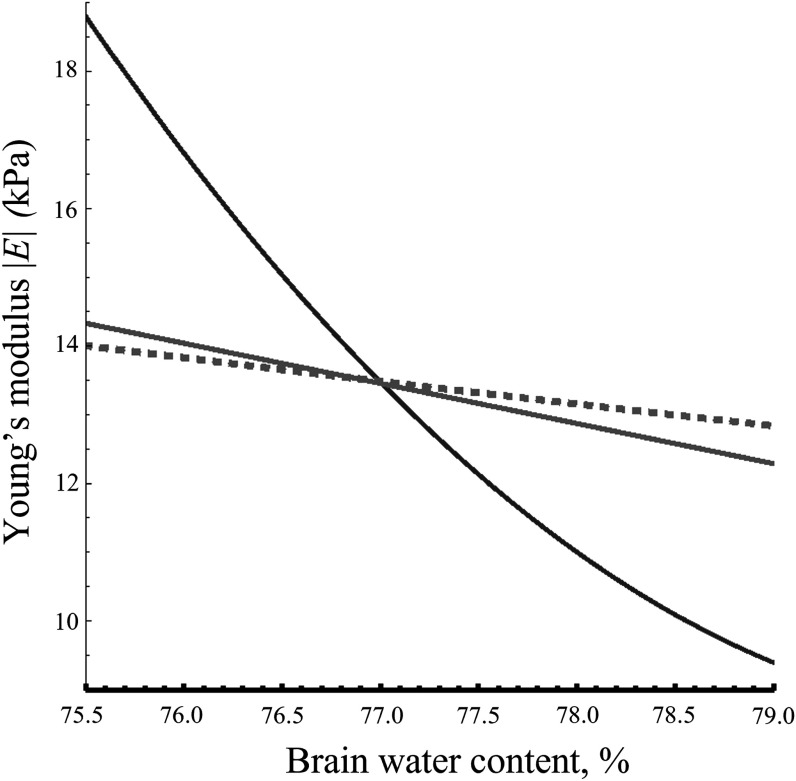
Stiffness as Young’s modulus in kPa (vertical) versus water content of the whole brain (percent). In red is the second order fit to the measured data at 2000 Hz shear waves across all mice. The blue dashed line is Christensen’s composite model considering all water content as supporting zero shear within an elastic matrix. In blue, solid line, is our simplified model, equation (7), where changes in elasticity and in water content are tied to the fluid dilation/constriction parameter $\chi $ that is set to unity at the reference value of 77% associated with young adult mice. The variation in $\chi $ in this example is tied to the global water content of the whole brain and not exclusively the glymphatic system as was the case in figure 5. Thus, these more general models that should apply to biphasic composites do not capture the strong correlation with age and fluid content found in the experimental data.

## Data Availability

File format incompatibilities and off-line storage create a significant time-and-effort requirement for distribution. The data that support the findings of this study are available upon reasonable request from the authors.

## Appendix. Alternative models with inadequate match to the measured results

To consider a range of rheological models, we first apply the landmark composite elastic material model proposed by Christensen (1969). Using a theory of strain energy, he derived a number of useful bounds on the macroscopic material properties of a composite. In particular, for the case of voids within an elastic matrix (water may be treated as void as it does not support shear waves) where the concentration of water is large (77% range in our case) and where the matrix (parenchymal) material is nearly incompressible with a Poisson’s ratio near 0.5, Christensen’s eqn (17) simplifies to:

\begin{eqnarray*}{G}_{{\mathrm{comp}}}=\displaystyle \frac{3}{5}G\left(1-V\right),\end{eqnarray*}

where *G*_comp_ is the composite shear wave modulus, *G* is the shear modulus of the matrix, and *V* is the fraction of the volume occupied by the fluid channels, assumed to be large, well above 50%. So, in this limit the shear modulus would approach zero as the fluid volume approaches 100%, and this final approach is linear. This is plotted in figure A1.

Alternatively, we return to the microchannel flow model where we assume that all components of the global brain fluid compartments have dilation or constriction by a factor of $\chi $ (not simply the glymphatic system as described in the results section). Thus equation (9) and its use in the earlier model is replaced by $ \% \,{\mathrm{water}}=77{\chi }^{3}$and the results are also shown in figure A1.

Both of these results indicate that more global models of fluid addition or subtraction to composite and biphasic channel models do not capture the steep trend linking brain stiffness to fluid percent measured in this study. Instead, assigning the changes to the glymphatic compartment and the elastic matrix create a combined effect of sufficient magnitude and functional dependence on changes in fluid percent exhibited in this study and seem to provide a reasonable basis for interpreting the measured trends.
